# The effect of radiation dose on mouse skeletal muscle remodeling

**DOI:** 10.2478/raon-2014-0025

**Published:** 2014-07-10

**Authors:** Justin P. Hardee, Melissa J. Puppa, Dennis K. Fix, Song Gao, Kimbell L. Hetzler, Ted A. Bateman, James A. Carson

**Affiliations:** 1 Department of Exercise Science, University of South Carolina, Columbia, SC, USA; 2 Departments of Biomedical Engineering and Radiation Oncology, University of North Carolina – Chapel Hill, Chapel Hill, NC, USA

**Keywords:** extracellular matrix, irradiation, oxidative metabolism, regeneration, skeletal muscle

## Abstract

**Background:**

The purpose of this study was to determine the effect of two clinically relevant radiation doses on the susceptibility of mouse skeletal muscle to remodeling.

**Materials and methods.:**

Alterations in muscle morphology and regulatory signaling were examined in tibialis anterior and gastrocnemius muscles after radiation doses that differed in total biological effective dose (BED). Female C57BL/6 (8-wk) mice were randomly assigned to non-irradiated control, four fractionated doses of 4 Gy (4x4 Gy; BED 37 Gy), or a single 16 Gy dose (16 Gy; BED 100 Gy). Mice were sacrificed 2 weeks after the initial radiation exposure.

**Results:**

The 16 Gy, but not 4x4 Gy, decreased total muscle protein and RNA content. Related to muscle regeneration, both 16 Gy and 4x4 Gy increased the incidence of central nuclei containing myofibers, but only 16 Gy increased the extracellular matrix volume. However, only 4x4 Gy increased muscle 4-hydroxynonenal expression. While both 16 Gy and 4x4 Gy decreased IIB myofiber mean cross-sectional area (CSA), only 16 Gy decreased IIA myofiber CSA. 16 Gy increased the incidence of small diameter IIA and IIB myofibers, while 4x4 Gy only increased the incidence of small diameter IIB myofibers. Both treatments decreased the frequency and CSA of low succinate dehydrogenase activity (SDH) fibers. Only 16 Gy increased the incidence of small diameter myofibers having high SDH activity. Neither treatment altered muscle signaling related to protein turnover or oxidative metabolism.

**Conclusions:**

Collectively, these results demonstrate that radiation dose differentially affects muscle remodeling, and these effects appear to be related to fiber type and oxidative metabolism.

## Introduction

The maintenance of skeletal muscle mass and metabolic function are critical for health.^1^ Skeletal muscle is a highly heterogeneous, plastic tissue that possesses varying metabolic and contractile properties. Muscle morphology, fiber type, and oxidative capacity can be influenced by many factors including its microenvironment, nutrient supply, and contractile activity.^1^ The application of radiation to skeletal muscle can alter the response to overload and impair regenerative processes.^2^–^5^ Radiation therapy is a common therapeutic modality for cancer^6^, and may contribute to muscular fatigue and weakness seen during treatment.^7^–^9^ Many of the biological effects of radiation therapy are tissue specific^10^, but the molecular mechanisms underlying tissue damage have not been clearly defined. Due to the post-mitotic state of skeletal muscle myofibers, basal muscle function has been commonly considered highly resistant to radiation.^11^,^12^ However, muscle contains many cell types that can influence the myofiber micro-environment to disrupt homeostasis. Therefore, understanding the adverse effects of radiation on skeletal muscle metabolism is needed to improve patient treatments and outcomes during radiation therapy.

Skeletal muscle mitochondria play an important role in metabolic health and myofiber function.^13^ Acute alterations to mitochondrial oxidative metabolism have been reported following therapeutic doses of radiation^14^, and can persist for up to 40 weeks following irradiation.^15^ Recent work suggests mitochondria may be susceptible to radiation, and may be a source of radiation-induced oxidative stress.^14^–^16^ Radiation increases the production of reactive oxygen and nitrogen species, which can result in oxidative damage to various cellular components. Specifically, radiation-induced free radical formation can result in protein oxidation, lipid peroxidation, and DNA damage.^17^ Skeletal myofibers vary in metabolic function and susceptibility to oxidative stress^18^,^19^, which may influence the muscles response to radiation. However, despite increased oxidative damage and impaired mitochondrial function following radiation^14^,^15^, the susceptibility of skeletal muscle to radiation-induced oxidative stress is poorly understood.

In addition to myofiber metabolic properties, the total radiation dose applied may be a significant variable in the disruption of muscle homeostasis. Higher radiation doses impair muscle regeneration^2^, attenuate overload-induced hyper-trophy^3^–^5^,^20^,^21^, and alter the structure and function of skeletal muscle.^11^,^12^,^22^–^24^ However, fractionating radiation into smaller doses attenuates skeletal muscle amino acid release when compared to a single larger dose.^25^ Despite the potential benefits of fractionation, lower radiation doses that are more clinically relevant can also impair skeletal muscle development^26^ and increase indices of muscle remodeling.^27^ Altered muscle plasticity following radiation exposure has been attributed to impaired satellite cell activity.^26^,^28^,^29^ Satellite cell and myoblast proliferation are reduced with as little as 2 Gy radiation exposure.^28^–^30^ These changes are accompanied by the induction of oxidative stress and apoptosis.^26^,^28^,^30^,^31^ Although there is evidence to suggest radiation dose can influence skeletal muscle remodeling, the role of skeletal muscle fiber type and metabolic capacity have not been determined.

The purpose of this study was to determine the effect of two clinically relevant radiation doses on the susceptibility of mouse skeletal muscle to remodeling. We hypothesized radiation would induce muscle remodeling in a dose dependent manner and that glycolytic type IIB muscle fibers would be more susceptible to radiation-induced changes in size and distribution, when compared to more oxidative type IIA muscle fibers. Alterations in muscle morphology and regulatory signaling were examined in female C57BL/6 mouse tibialis anterior and gastrocnemius muscles after radiation doses that differed in total biological effective dose (BED). Mice were exposed to radiation with a single (16 Gy; BED 100 Gy) or fractionated (4x4 Gy; BED 37 Gy) doses over a period of 2 weeks.

## Materials and methods

### Animals

Twenty-four female C57BL/6 mice were purchased from Jackson Laboratory (Bar Harbor, ME) and housed at the animal resource facility at the University of North Carolina-Chapel Hill. Mice were grouped housed, given access to food and water *ad libitum*, and kept on a 12-hour light-dark cycle. The study was approved by the Institutional Animal Care and Use Committee at the University North Carolina-Chapel Hill and was carried out in compliance with the Guide for the Care and Use of Laboratory Animals (National Institutes of Health, Bethesda, MD).

### Experimental design

**FIGURE 1.**
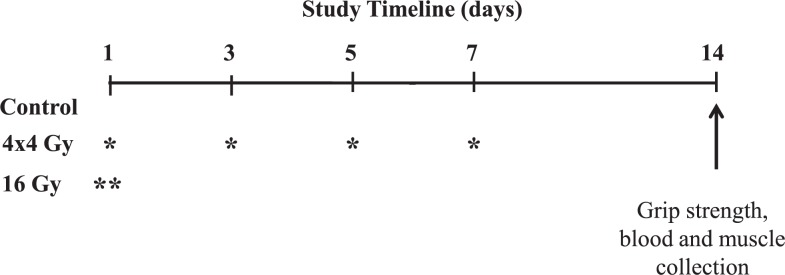
Experimental Design. At 8-weeks of age twenty-four female C57BL/6 mice were randomly assigned to non-irradiated control, four fractionated doses of 4 Gray (4x4 Gy; BED 37 Gy), or a single 16 Gy dose (16 Gy; BED 100 Gy) based on the total biological effective dose (BED). Mice were sacrificed 2-weeks after the first radiation exposure. All mice underwent sham or radiation exposure starting on day 1. Mice receiving fractionated doses (4x4 Gy) were irradiated every other day over a period of 2 weeks. Mice receiving a single radiation (16 Gy) were only irradiated on day 1. Grip strength was performed in the morning prior to sacrifice. ^*^ = 4 Gy radiation exposure; ^**^ = 16 Gy radiation exposure.

At 8 weeks of age, mice were randomly assigned (n=8/group) to non-irradiated control, four fractionated doses of 4 Gray (4x4 Gy; BED 37 Gy), or a single 16 Gy dose (16 Gy; BED 100 Gy) based on the total biological effective dose (BED). Mice in the 16 Gy treatment received a single radiation dose on day 1. Mice in the 4x4 Gy treatment were exposed to fractionated radiation doses every other day over a period of two weeks (Figure 1). This dosing strategy was designed to model normal tissue radiation exposure during the treatment of pelvic malignancies such as cervical cancer when treated with external beam radiotherapy, where muscles in each hip would be exposed to approximately half (up to 0.9 Gy per fraction) of the total dose that the tumor receives (54 Gy in 30x1.8 Gy fractions). Specifically, the BED was calculated using the Fowler equation, accounting for dose-per-fraction and relative tissue sensitivity (α/β = 3). The calculated BED for the fractionated and single dosing strategies of mouse-limb exposures are 37 Gy and 100 Gy, respectively. By comparison, the BED exposure to each human-hip during treatment (30x0.9 Gy) is 35 Gy.^32^ Mi ce were sacrificed 2-weeks following the first radiation exposure. Thi s time-point was chosen to examine the acute effects that may develop following radiation exposure.^33^

### Hindlimb irradiation

Radiation to the hindlimbs was performed as previously described, with slight modifications.^27^ Animals were anesthetized with 4% isoflurane and placed in an irradiator device (X-RAD 320, North Branford, CT). Sedation was maintained with constant 2.5% isoflurane administration throughout the procedure. The collimator was adjusted so the radiation field included only the region distal to the pelvis. Irradiation was performed with 320 kV X-rays at a dose rate of 0.5 Gy/min for a total BED of 37 and 100 Gy. Control mice underwent the same procedure without radiation exposure.

### Hindlimb grip strength

Prior to sacrifice, hindlimb grip strength was measured with the Grip Tester (Model 1027CSM; Columbus Instruments, Columbus, OH) with slight modifications as previously described.^34^ Mice were held with hindlimbs positioned on a horizontal grid connected to a force transducer. Mice were then pulled away from the grid until they could no longer maintain grip. Each mouse underwent two sets of five repetitions of force measurements with two minutes rest between each set. The highest and lowest force measurements from each set were removed and an average force measurement for each mouse was calculated.

### Tissue collection

At the time of sacrifice, mice were euthanized by cervical dislocation. Hindlimb skeletal muscles were excised, snap-frozen in liquid nitrogen, and stored at −80°C until analysis. The tibia and femur were removed, cleaned of all soft tissue, and stored in ethanol until analysis.

### Total muscle protein and RNA content

Total muscle protein and RNA content of the gastrocnemius muscle was determined according to Fleck and Munro as previously described.^35^,^36^ Briefly, frozen muscle samples (∼20 mg) were homogenized in 0.2M HClO_4_ and centrifuged (4°C at 12,000 x g for 10 minutes). Following two washes in 0.2M HClO_4_, the remaining pellet was air dried, suspended in 0.3M KOH and incubated at 37°C overnight. An aliquot was removed and total protein concentration was determined by the Bradford method.^37^ To the remaining sample, 1.2M HClO_4_ was added and centrifuged for 10 minutes at 4°C (12,000 x g). After centrifugation, the supernatant was removed and the pellet was washed twice with 0.2M HClO_4_, with subsequent supernatants removed and pooled with previous washes. Absorbance was read at 260 nm to determine total RNA concentration.

### Tibialis anterior morphology

Transverse muscle sections (∼10 μm) were cut from the mid-belly of the tibialis anterior (TA) on a cryostat at −20°C and stored at −80°C until further analysis. Hematoxylin and eosin (H&E) staining was performed on cross-sections to determine myofiber morphology as described previously.^27^,^36^ H&E stained muscle sections were digitized at 400X magnification and analyzed using a computer with ImageJ imaging software (NIH, Bethesda, MD). Centralized nuclei, defined as a nuclei found equidistant from a well-defined sarcolemma, were quantified from these images, and were expressed as the percent of centralized nuclei per total muscle fibers. The extracellular matrix area was quantified as previously described.^38^ Images containing well-defined sarcolemma were traced and the extracellular matrix is expressed as the percentage of whole muscle.

### Immunohistochemistry for myosin heavy chain type IIA and IIB

Immunohistochemistry for myosin heavy chain type IIA and IIB was performed as previously described.^39^ Transverse sections of the TA were air dried for 10 minutes, fixed in cold acetone for 1 minute, and washed in PBS for 5 minutes. Sections were quenched in 0.3% H_2_O_2_-methanol solution for 20 minutes and rinsed in PBS for 5 minutes three times. Sections were blocked in 10% normal goat serum (Vectastain ABC kit, Vector Laboratories, Burlingame, CA) in PBS for 1 h at room temperature and then incubated overnight at 4°C with primary antibodies (SC-71 for type IIA; BF-F3 for type IIB; Iowa Hybridoma Bank). Sections were then washed three times for 5 minutes in PBS. Secondary antibodies (Vector Laboratories) were applied to the sections for 1 h at 37 °C and sections were washed again three times for 5 minutes in PBS. Avidin-biotin complex system (ABC; Vector Laboratories) was used to detect the biotinylated secondary antibody by incubating sections in ABC solution at room temperature for 30 min. Sections were washed three times for 5 minutes in PBS and visualized by incubating in DAB solution for 6 minutes (Vectastain DAB kit, Vector Laboratories, Burlingame, CA). The sections were rinsed in dH_2_O, dried, and mounted by cover glasses with a mounting media. Muscle sections were digitized and analyzed ImageJ imaging software. The percentage of type IIA and IIB was quantified and is expressed as the percent per total muscle fibers. Fiber-type specific cross-sectional area (CSA) was quantified by an investigator blinded to each group.

### Succinate dehydrogenase activity

Succinate dehydrogenase (SDH) enzyme activity was performed as previously described to determine muscle oxidative capacity.^39^ Briefly, frozen cross-sections were air dried for 10 minutes, followed by incubation in a solution containing 0.2M phosphate buffer (pH 7.4), 0.1M MgCl_2_, 2.4 mM nitroblue tetrazolium (NBT), and 0.2M succinate acid for 45 minutes at 37°C. Sections were then washed in dH_2_O for 3 minutes, dehydrated in 50% ethanol for 2 minutes, and mounted for viewing with mounting media. Digital photographs were taken from each section at 200X magnification with a Nikon spot camera, and fibers were traced with ImageJ imaging software (∼150 per animal). The intensity of SDH staining activity was determined by subtracting the background from each slide to create an integrated optical density for each myofiber. Based on the optical density fibers were classified as light or dark stained. The percentage of each stain was quantified and was expressed as a percent per total muscle fibers. Myofiber CSA was quantified in dark and light stained fibers.

### Western blot analysis

Western blot analysis was performed as previously described.^36^,^39^ Briefly, frozen gastrocnemius muscle was homogenized, and protein concentration was determined by the Bradford method.^37^ Crude muscle homogenates (15–40 μg) were fractionated on 8–15% polyacrylamide gels. Gels were transferred to polyvinylidene difluoride membranes overnight at 4°C. Equal protein loading of the gels was assessed by Ponceau staining. Membranes were then blocked in 5% milk-TBST for one hour at room temperature. Primary antibodies for 4-hydroxynonenal (4-HNE; Alpha Diagnostics), ubiquitin, phosphorylated (T202/Y204) and total ERK1/2, phosphorylated (T180/Y182) and total p38, phosphorylated (S473 and T308) and total Akt, phosphorylated (S253) and total FOXO3A, Atrogin-1, LC3B, PGC-1α (Santa Cruz Biotechnology), mitochondria transcription factor A (TFAM), NADH-ubiquinone oxidoreductase 75 kDa subunit (NDUFS1), cytochrome c, glyceralde-hyde 3-phosphate dehydrogenase (GAPDH) were incubated at 1:2000 to 1:10,000 dilutions in 5% milk-TBST overnight at 4°C. Secondary anti-rabbit and anti-mouse IgG conjugated antibodies were incubated with membranes at a 1:2000 dilution in 5% milk-TBST for 2 hours at room temperature. All antibodies were purchased from Cell Signaling unless otherwise stated. Enhanced chemiluminescence was used to visualize the antibody-antigen interaction and developed by autoradiography (Kodak, Biomax). Immunoblots were digitally scanned and analyzed by measuring the integrated optical density of each band using ImageJ imaging software.

### Slot blot analysis

The slot blot technique was performed to detect lipid peroxidation and protein ubiquitination in muscle as previously described.^39^ Briefly, graded quantities (10, 20, and 30μg) of crude muscle homogenates were transferred to a PVDF membrane using the Bio-Dot SF microfiltration apparatus (Bio-Rad, Hercules, CA) following the manufacturer’s instructions. The membrane was then probed and analyzed as described above (Western blot analysis).

### Statistical Analysis

Results are reported as means ± standard error of the mean. Comparisons between treatment groups were assessed by one-way analysis of variance (ANOVA). Post hoc analyses were performed with the Tukey’s multiple comparison test when appropriate. Frequency histograms and the percentage of small and large myofibers were compared by Chi-square analysis. Significance was set at *P*<.05. Statistical analysis was performed using SigmaStat version 3.5 (Systat Software Inc., Richmond, CA).

## Results

### Body weight, hindlimb grip strength, muscle mass & total muscle protein and RNA content

At 8 weeks of age twenty-four female C57BL/6 mice were subjected to radiation or sham procedures and were sacrificed 2 weeks following the first exposure (Figure 1). Neither radiation treatment altered overall body weight, hindlimb grip strength, and tibialis anterior muscle mass (Table 1). There was also no radiation effect on gastrocnemius muscle mass (Table 2). However, the 16 Gy treatment decreased total gastrocnemius protein 35% and RNA content 20% when compared to control muscle (Table 2). In contrast, gastrocnemius total protein and RNA content was not altered by the 4x4 Gy treatment.

### Tibialis anterior morphology

**FIGURE 2.**
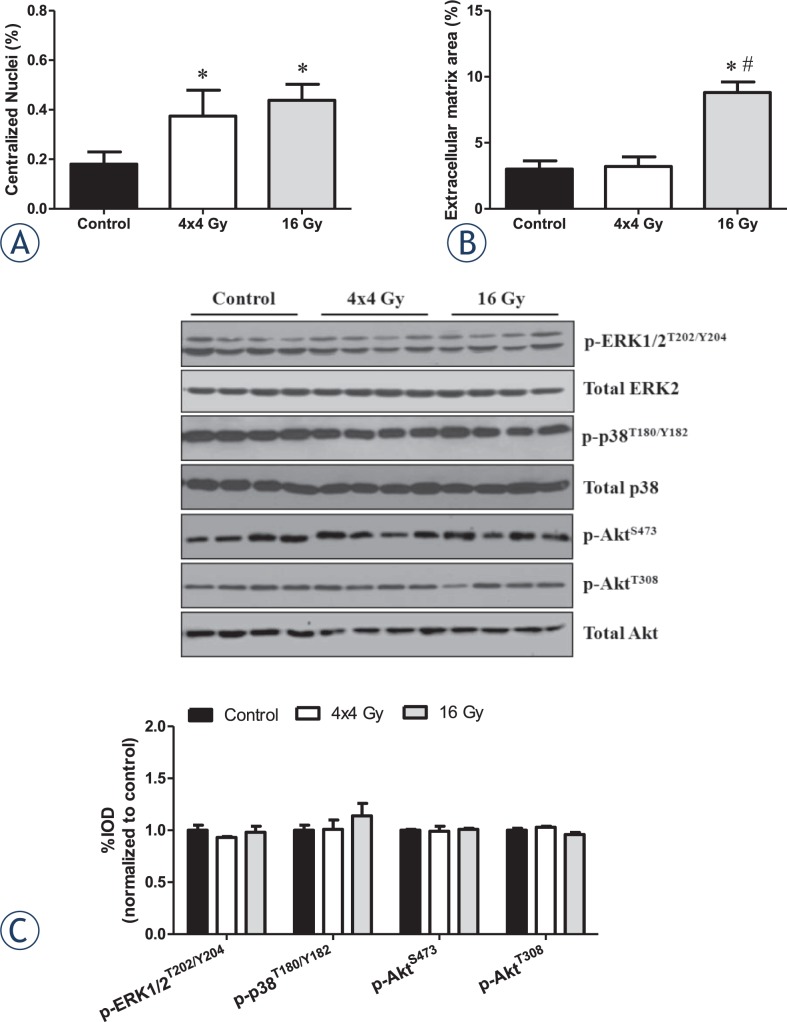
Effects of hindlimb irradiation on muscle regeneration and remodeling. A The percentage of myofibers containing centralized nuclei in the tibialis anterior muscle. Centralized nuclei were defined as nuclei found at equidistance from a well-defined sarcolemma and are expressed as the percent of centralized nuclei per total muscle fibers. B The percentage of extracellular matrix area in the tibialis anterior muscle. Extracellular matrix is expressed as the percent of extracellular matrix per total muscle area. C *Upper.* Representative immunoblot of phosphorylated and total forms of ERK1/2, p38, and Akt proteins in the gastrocnemius muscle. *Lower.* Quantification of phospho protein activation (ERK1/2, p38, and Akt) is shown as the ratio of phosphorylated to total protein expression. Values are means ± standard error. Statistical significance was set at *P*<.05. ^*^ = statistically different from control; # = statistically different from 4x4 Gy.

The percentage of centralized nuclei and extracellular matrix (ECM) area was quantified to examine characteristics of muscle remodeling. Both radiation treatments increased the percentage of myofibers containing centralized nuclei (Figure 2A). Only the 16 Gy treatment increased the percentage of ECM when compared to control muscle (Figure 2B). No changes were observed with 4x4 Gy treatment (Figure 2B). Neither treatment altered muscle signaling related to remodeling and growth (Figure 2C).

### Skeletal muscle oxidative stress and protein turnover

**FIGURE 3.**
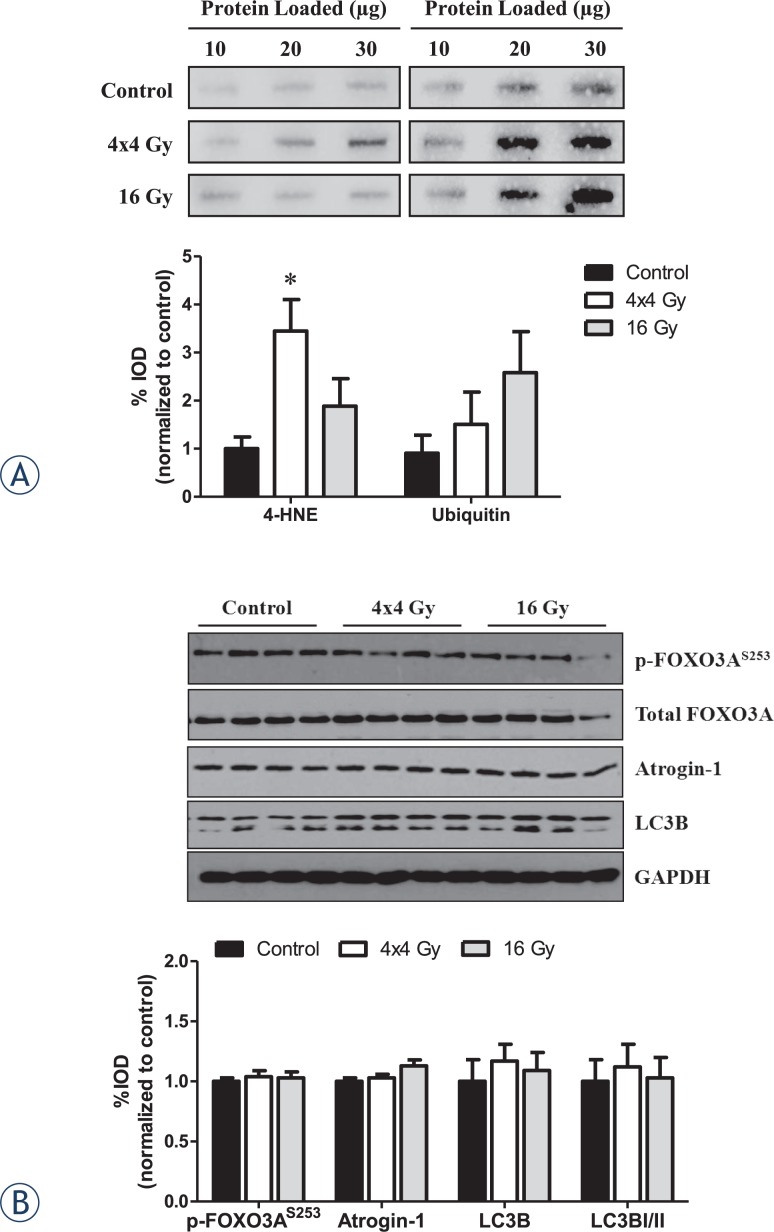
Effects of hindlimb irradiation on gastrocnemius muscle oxidative stress and protein turnover. A *Upper left.* Representative immunoblot of 4HNE protein expression. *Lower left.* Quantification of 4HNE protein expression. *Upper right.* Representative immunoblot of ubiquitin protein expression. *Lower right.* Quantification of ubiquitin protein expression. B *Upper.* Representative immunoblot of phosphorylated and total FOXO3A, and total Atrogin-1, LC3B and GAPDH protein expression. *Lower.* Quantification of phosphorylated and total FOXO3A, and total Atrogin-1, LC3B and GAPDH proteins. Values are normalized to control and are presented as means ± standard error. Statistical significance was set at *P*<.05. ^*^ = statistically different from control; 4-HNE = 4-hydroxynonenal; μg = microgram; Gy = gray.

We examined the effects of radiation dose on muscle oxidative stress and protein turnover in the gastrocnemius muscle. The 4x4 Gy treatment increased the expression of 4-hydroxynonenal (4-HNE) protein, a marker of oxidative stress, while no change was found with the 16 Gy treatment (Figure 3A). Ubiquitination of proteins was not significantly altered by either radiation treatment. However, the 16 Gy treatment demonstrated a highly variable response (*P*=.10; Figure 3A). Neither treatment altered muscle signaling related to protein turnover and autophagy (Figure 3B).

### Tibialis anterior type IIA and IIB fiber-type

**FIGURE 4.**
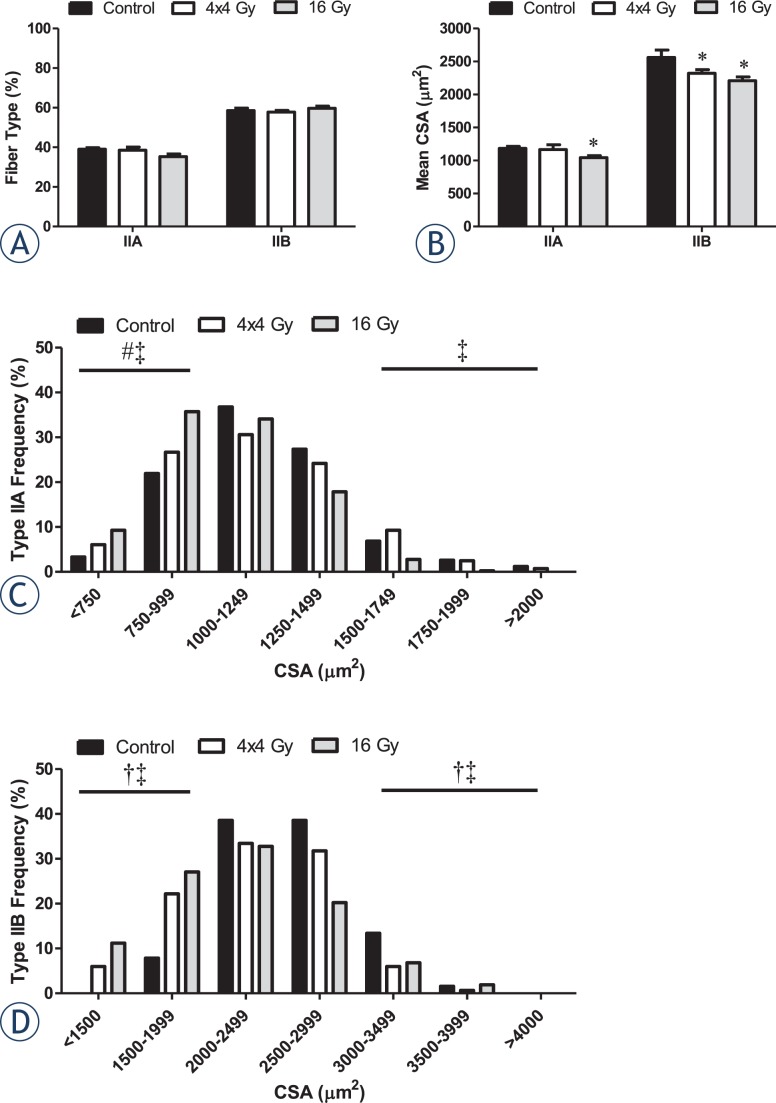
Effects of hindlimb irradiation on tibialis anterior myosin heavy chain IIA and IIB expression and size. A The percentage of type IIA and IIB myofibers. B Mean cross-sectional area (CSA) of type IIA and IIB myofibers. C Mean CSA distribution of type IIA myofibers. D Mean CSA distribution of type IIB myofibers. The frequencies of small and large myofibers (±2SD of mean) were compared by Chi-square analysis. Values are means ± standard error. Statistical significance was set at *P*<.05. Black box, Control. White box, 4x4 Gy. Grey box, 16 Gy. μm = micrometer; Gy = gray. ^*^ = statistically different from control; † = statistical difference between control and 4x4 Gy; ‡ = statistical difference between control and 16 Gy; # = statistical difference between 4x4 Gy and 16 Gy.

Type IIA and IIB fiber incidence and size distribution were examined in the tibialis anterior (TA) muscle of control and irradiated mice. Neither radiation treatment altered the overall percentage of type IIA and IIB fibers in the muscle (Figure 4A). Only the 16 Gy treatment decreased type IIA myofiber cross-sectional area (CSA) (Figure 4B) and increased the incidence of small diameter IIA myofibers when compared to the control and 4x4 Gy treatments (Figure 4C). In contrast, both radiation treatments decreased type IIB myofiber mean CSA (Figure 4B), which was accompanied by an increased incidence of small diameter myofibers (Figure 4D).

### Skeletal muscle oxidative capacity

**FIGURE 5.**
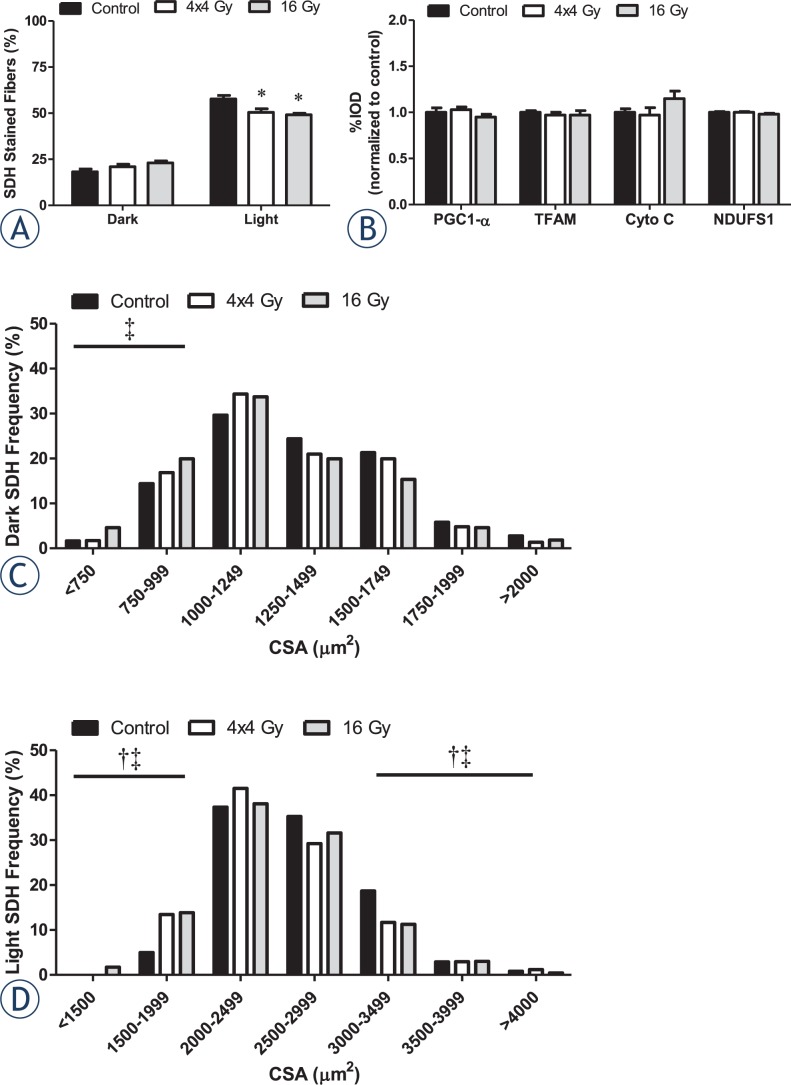
Effects of hindlimb irradiation on muscle oxidative capacity. A The percentage of dark and light SDH stained myofibers in the tibialis anterior muscle. B Quantification of total forms of PGC-1α, TFAM, cytochrome C, and NDUFS1 proteins in the gastrocnemius muscle. C Mean cross-sectional area (CSA) distribution of dark SDH stained myofibers in the tibialis anterior muscle. D Mean CSA distribution of light SDH stained myofibers in the tibialis anterior muscle. The frequencies of small and large myofibers (±2SD of mean) were compared by Chi-square analysis. Values are means ± standard error. Statistical significance was set at *P*<.05. Black box, Control. White box, 4x4 Gy. Grey box, 16 Gy. μm = micrometer; Gy = gray; ^*^ = statistically different from control; † = statistical difference between control and 4x4 Gy; ‡ = statistical difference between control and 16 Gy.

Succinate dehydrogenase activity (SDH) in myofibers was examined as an indicator of myofiber oxidative metabolic capacity.^39^ Neither radiation treatment altered the percentage of high SDH activity fibers found in the muscle (*P*=.07; Figure 5A). In contrast, both radiation treatments decreased the percentage of low SDH activity fibers (Figure 5A). Neither radiation treatment altered muscle protein expression related to mitochondrial biogenesis and oxidative metabolism (Figure 5B). Only the 16 Gy treatment increased the incidence of small myofibers with high SDH activity (Figure 5C), while both radiation treatments increased the incidence of small myofibers exhibiting low SDH activity (Figure 5D).

## Discussion

Despite recent advances in radiation treatment to minimize exposure to surrounding normal tissues, treated patients still display muscular fatigue and weakness.^8^ Additionally, radiation exposure can directly alter the response of rodent skeletal muscle to overload and injury.^11^,^12^,^22^,^23^ While radiation is thought to be detrimental to skeletal muscle function, the biological basis of these radiation-induced responses has not been clearly delineated. Furthermore, the ability of radiation dose alone to induce skeletal muscle remodeling has not been clearly defined. We report the novel finding that radiation dose can differentially affect muscle morphology and oxidative damage. Our results demonstrate that both radiation treatments increase muscle remodeling as indicated by the prevalence on central nuclei containing fibers, but only the 16 Gy treatment increased the muscle’s extracellular matrix volume. Conversely, only the 4x4 Gy treatment increased muscle oxidative damage. Additionally, radiation-induced alterations to myofiber size were affected by fiber type and fiber oxidative capacity. None of these changes were associated with altered protein expression related to mitochondrial biogenesis, oxidative metabolism or signaling involved in the regulation of skeletal muscle mass. Collectively, these results demonstrate that radiation dose differentially affects muscle remodeling, and these affects are impacted by fiber type and oxidative metabolism.

We provide morphological evidence that radiation dose is a critical element for the induction of skeletal muscle remodeling. High and low radiation doses have been shown to attenuate overload and normal maturation induced muscle growth.^3^–^5^,^20^,^26^ Regardless of the BED there was an increase in myofibers exhibiting centralized nuclei, an indicator of muscle regeneration. Similar findings have also been reported in response to lower radiation doses.^27^ In this study, a larger BED resulted in an expansion of the extracellular matrix area and was accompanied by a decrease in total protein and RNA content. The decrease in total protein content may therefore be related to muscle remodeling. The release of skeletal muscle amino acids that occurs as a result of muscle proteolysis is thought to occur in several muscle wasting conditions.^1^,^40^ Altman and Schwenen^7^ demonstrated increased release of amino acids 4–6 hours following high dose radiation, and was this response was attenuated by fractionating into smaller doses.^25^ However, muscle morphology was not examined in these studies. We report that neither radiation dose was able to alter muscle mass or volitional grip strength. Thus, it appears that neither radiation treatment influenced normal muscle growth over the 2-week experimental period. In support of this, we found no radiation-induced changes in muscle signaling related to muscle growth, including Akt and FOXO signaling pathways. Future work is needed to understand the associations between altered protein content and muscle remodeling in response to radiation.

Our results demonstrate radiation can induce fiber-type specific alterations in size and distribution that are related to the total BED. Regardless of the dose applied, decreased type IIB myofiber CSA was accompanied by an increase in small fiber incidence. Similar findings have been reported in the extensor digitorum longus of mice and rats exposed to a single radiation dose.^3^,^4^ Intermediate (type IIA/IIX) and glycolytic fiber (type IIB) size was decreased in mice^3^, and in all rat fiber types (type I, IIA, IIX, and IIB).^4^ In addition, myofibrillar degeneration and myofiber atrophy in type II fibers occurred following x-ray irradiation in rabbit pectoralis major muscle, but specific isoforms were not quantified.^11^ Type IIB myofibers are more susceptible to free radical formation and oxidative stress^18^,^19^, which may account for the radiation-induced disruption of muscle homeostasis. Interestingly, we found the increase in small type IIA myofiber incidence was attenuated by the use of a lower dose. Further, changes in small fiber incidence were independent of an overall fiber type shift, which is consistent with previous reports.^3^,^4^ It has been suggested that a minimal radiation dose is needed to induce skeletal muscle proteolysis^7^,^25^; however, specific myofiber alterations were not examined in these studies. Our findings provide evidence that fiber type can interact with radiation in a dose-dependent manner, and this relationship has a significant impact on radiation-induced muscle remodeling. Further research is needed to determine the cellular signals underlying this relationship.

There is currently a limited understanding of how radiation affects myofiber metabolic properties. Mitochondria are a likely target of radiation for the induction of cellular dysfunction that leads to tissue damage.^16^ Since oxidative capacity and mitochondrial content can vary by muscle fiber phenotype, these properties may influence the radiation sensitivity of myofibers. Similar to our fiber type analysis, both radiation doses in our study decreased the size of myofibers with low oxidative capacity, but only the 16 Gy dose decreased the size of myofibers with high oxidative capacity. Thus, there were differential responses related to the muscle fiber’s metabolic properties and the radiation dose applied. Skeletal muscle irradiation increases acute oxidative stress and alter proteins related to contractile function and energy metabolism.^41^ Our results support differential responses to radiation-induced oxidative stress which may be dependent on the muscles oxidative capacity. We found evidence of oxidative stress, as indicated by increased 4-HNE protein expression, which persisted for a week after the last radiation exposure. Interestingly, the 4x4 Gy dose had greater 4-HNE protein expression, indicating that this may be more dependent on the timing of the last radiation exposure rather than the total dose applied. Cardiac muscle cells have demonstrated radiation-induced alterations to mitochondrial function.^14^,^15^ A clinically relevant dose (2 Gy) was sufficient to increase cardiac myocyte mitochondrial protein oxidation, and resulted in decreased mitochondrial respiration and protein expression four weeks after radiation exposure.^14^ Similar mitochondrial impairments were also observed 40 weeks following acute radiation.^15^ These results demonstrate the potential for acute radiation to induce long-term mitochondrial dysfunction.^14^,^15^ Future research is needed to determine if these responses occur in skeletal muscle.

In summary, we demonstrate dose dependent radiation-induced muscle remodeling related to expansion of the extracellular matrix and oxidative stress. The effect of radiation dose on myofiber size was affected by fiber type and oxidative metabolism. Our results demonstrate that type IIB, glycolytic myofibers were susceptible to radiation-induced changes in myofiber size regardless of the total dose. However, type IIA oxidative myofiber size was not affected by the lower radiation dose. The current findings provide rationale for further examination of how radiation dose interacts with muscle fiber type and oxidative capacity to alter the remodeling response. The presence of differential responses to various radiation doses may have important clinical ramifications for the maintenance of muscle mass and function in individuals undergoing radiation treatment. Future research is needed to determine long-term functional outcomes following radiation to skeletal muscle.

## Figures and Tables

**TABLE 1.** Body weight, hindlimb grip strength, and tibialis anterior muscle mass at sacrifice in control and irradiated mice

**Treatment**	**n**	Body weight (g, AM ± SE)^*^	**Grip strength (N, AM ± SE)**	**TA mass (mg, AM ± SE)**	**TA:BW (AM ± SE)**
Control	8	21.9 ± 0.3	0.70 ± 0.03	37 ± 1.4	1.7 ± 0.06
4x4 Gy	8	22.0 ± 0.3	0.67 ± 0.03	37 ± 1.0	1.7 ± 0.04
16 Gy	8	22.2 ± 0.2	0.67 ± 0.03	35 ± 0.6	1.6 ± 0.04

n = number of animals;

* AM ± SE = means ± standard error; N = newtons; TA = tibialis anterior; TA:BW = TA normalized to body weight; g = grams; mg = milligrams; Gy = Gray

**TABLE 2.** Gastrocnemius muscle mass, total protein and RNA content in control and irradiated mice.

**Treatment**	**n**	**Gastroc mass (mg, AM ± SE)**	**Protein (mg/muscle, AM ± SE)**	**RNA (mg/muscle, AM ± SE)**	**Protein/RNA (AM ± SE)**
Control	6	101 ± 1.3	13.9 ± 1.6	126 ± 3	0.11 ± 0.01
4x4 Gy	7	102 ± 2.5	10.3 ± 1.6	116 ± 9	0.09 ± 0.02
16 Gy	6	98 ± 1.4	9.1 ± 0.5^*^	101 ± 10^*^	0.09 ± 0.01

n = number of animals; AM ± SE = means ± standard error; Gastroc = gastrocnemius; mg = milligram; Gy = Gray;

* = statistically different from Control

## References

[1] Wolfe RR (2006). The underappreciated role of muscle in health and disease. Am J Clin Nutr.

[2] Gulati AK (1987). The effect of X-irradiation on skeletal muscle regeneration in the adult rat. J Neurol Sci.

[3] Rosenblatt JD, Parry DJ (1992). Gamma irradiation prevents compensatory hypertrophy of overloaded mouse extensor digitorum longus muscle. J Appl Physiol.

[4] Rosenblatt JD, Parry DJ (1993). Adaptation of rat extensor digitorum longus muscle to gamma irradiation and overload. Pflugers Arch.

[5] Rosenblatt JD, Yong D, Parry DJ (1994). Satellite cell activity is required for hypertrophy of overloaded adult rat muscle. Muscle Nerve.

[6] Zachariah B, Balducci L, Venkattaramanabalaji GV, Casey L, Greenberg HM, DelRegato JA (1997). Radiotherapy for cancer patients aged 80 and older: a study of effectiveness and side effects. Int J Radiat Oncol Biol Phys.

[7] Altman KI, Schwenen M (1987). Increased catabolism of muscle proteins as a manifestation of radiation myopathy. Radiat Environ Biophys.

[8] Giacalone A, Quitadamo D, Zanet E, Berretta M, Spina M, Tirelli U (2013). Cancer-related fatigue in the elderly. Support Care Cancer.

[9] Kurohara SS, Rubin P, Hempelmann LH (1961). Creatinuria and fatigue in patients undergoing radiation therapy. Radiology.

[10] Denekamp J, Rojas A (1989). Cell kinetics and radiation pathology. Experientia.

[11] Khan MY (1974). Radiation-induced changes in skeletal muscle. An electron microscopic study. J Neuropathol Exp Neurol.

[12] Lewis RB (1954). Changes in striated muscle following single intense doses of x-rays. Lab Invest.

[13] Nunnari J, Suomalainen A (2012). Mitochondria: in sickness and in health. Cell.

[14] Barjaktarovic Z, Schmaltz D, Shyla A, Azimzadeh O, Schulz S, Haagen J (2011). Radiation-induced signaling results in mitochondrial impairment in mouse heart at 4 weeks after exposure to X-rays. PLoS One.

[15] Barjaktarovic Z, Shyla A, Azimzadeh O, Schulz S, Haagen J, Dorr W (2013). Ionising radiation induces persistent alterations in the cardiac mitochondrial function of C57BL/6 mice 40 weeks after local heart exposure. Radiother Oncol.

[16] Kam WW, Banati RB (2013). Effects of ionizing radiation on mitochondria. Free Radic Biol Med.

[17] Azimzadeh O, Scherthan H, Sarioglu H, Barjaktarovic Z, Conrad M, Vogt A (2011). Rapid proteomic remodeling of cardiac tissue caused by total body ionizing radiation. Proteomics.

[18] Anderson EJ, Neufer PD (2006). Type II skeletal myofibers possess unique properties that potentiate mitochondrial H(2)O(2) generation. Am J Physiol Cell Physiol.

[19] Feng J, Xie H, Meany DL, Thompson LV, Arriaga EA, Griffin TJ (2008). Quantitative proteomic profiling of muscle type-dependent and age-dependent protein carbonylation in rat skeletal muscle mitochondria. J Gerontol A Biol Sci Med Sci.

[20] Adams GR, Caiozzo VJ, Haddad F, Baldwin KM (2002). Cellular and molecular responses to increased skeletal muscle loading after irradiation. Am J Physiol Cell Physiol.

[21] Phelan JN, Gonyea WJ (1997). Effect of radiation on satellite cell activity and protein expression in overloaded mammalian skeletal muscle. Anat Rec.

[22] Bergstrom RM, Salmi A (1962). Radiation-induced damage in the ultrastructure of striated muscle. Exp Cell Res.

[23] Darden EB (1960). Changes in membrane potentials, K content, and fiber structure in irradiated frog sartorius muscle. Am J Physiol.

[24] Wernig A, Zweyer M, Irintchev A (2000). Function of skeletal muscle tissue formed after myoblast transplantation into irradiated mouse muscles. J Physiol.

[25] Schwenen M, Altman KI, Schroder W (1989). Radiation-induced increase in the release of amino acids by isolated, perfused skeletal muscle. Int J Radiat Biol.

[26] Olive M, Blanco R, Rivera R, Cinos C, Ferrer I (1995). Cell death induced by gamma irradiation of developing skeletal muscle. J Anat.

[27] Bandstra ER, Thompson RW, Nelson GA, Willey JS, Judex S, Cairns MA (2009). Musculoskeletal changes in mice from 20–50 cGy of simulated galactic cosmic rays. Radiat Res.

[28] Caiozzo VJ, Giedzinski E, Baker M, Suarez T, Izadi A, Lan M (2010). The radiosensitivity of satellite cells: cell cycle regulation, apoptosis and oxidative stress. Radiat Res.

[29] Cho-Lim JJ, Caiozzo VJ, Tseng BP, Giedzinski E, Baker MJ, Limoli CL (2011). Satellite cells say NO to radiation. Radiat Res.

[30] Jurdana M, Cemazar M, Pegan K, Mars T (2013). Effect of ionizing radiation on human skeletal muscle precursor cells. Radiol Oncol.

[31] Latella L, Lukas J, Simone C, Puri PL, Bartek J (2004). Differentiation-induced radioresistance in muscle cells. Mol Cell Biol.

[32] Fowler JF (2010). 21 years of biologically effective dose. Br J Radiol.

[33] Nagler RM (2001). Extended-term effects of head and neck irradiation in a rodent. Eur J Cancer.

[34] Puppa MJ, White JP, Velazquez KT, Baltgalvis KA, Sato S, Baynes JW (2012). The effect of exercise on IL-6-induced cachexia in the Apc (Min/+) mouse. J Cachexia Sarcopenia Muscle.

[35] Fleck A, Munro HN (1962). The precision of ultraviolet absorption measurements in the Schmidt-Thannhauser procedure for nucleic acid estimation. Biochim Biophys Acta.

[36] Mehl KA, Davis JM, Berger FG, Carson JA (2005). Myofiber degeneration/regeneration is induced in the cachectic ApcMin/+ mouse. J Appl Physiol.

[37] Bradford MM (1976). A rapid and sensitive method for the quantitation of microgram quantities of protein utilizing the principle of protein-dye binding. Anal Biochem.

[38] Huang Y, de Boer WB, Adams LA, Macquillan G, Rossi E, Rigby P (2013). Image analysis of liver collagen using sirius red is more accurate and correlates better with serum fibrosis markers than trichrome. Liver Int.

[39] White JP, Baltgalvis KA, Puppa MJ, Sato S, Baynes JW, Carson JA (2010). Muscle oxidative capacity during IL-6-dependent cancer cachexia. Am J Physiol Regul Integr Comp Physiol.

[40] Tisdale MJ (2002). Cachexia in cancer patients. Nat Rev Cancer.

[41] Fedorova M, Kuleva N, Hoffmann R (2009). Reversible and irreversible modifications of skeletal muscle proteins in a rat model of acute oxidative stress. Biochim Biophys Acta.

